# Performance Comparison of Transition Metal (Cr, Mn, Fe, Co, Ni, Cu)-Fluoride Conversion Cathodes in Thin-Film Solid-State Batteries

**DOI:** 10.1021/acsaem.5c01772

**Published:** 2025-10-03

**Authors:** Joel Casella, Jȩdrzej Morzy, Felix C. Mocanu, Arnold Müller, Maksym Yarema, Moritz H. Futscher, M. Saiful Islam, Yaroslav E. Romanyuk

**Affiliations:** † Laboratory for Thin Films and Photovoltaics, 548513 Empa—Swiss Federal Laboratories for Material Science and Technology, Dübendorf 8600, Switzerland; ‡ Energy Materials Research Group, Department of Materials, 28501 University of Oxford, Oxford OX1 3PH, United Kingdom; § Laboratory of Ion Beam Physics, 170993 ETH Zürich, Zürich 8093, Switzerland; ∥ 27219 Institute for Electronics, Department of Information Technology and Electrical Engineering, ETH, Zurich 8092, Switzerland

**Keywords:** li-ion battery, solid-state battery, thin-film, iron fluoride, conversion cathode, LiPON, battery, transition metals

## Abstract

Transition metal fluorides (TMFs) are promising alternatives for Li battery cathode active materials as they can show specific capacities up to 619 mAh/g for CrF_3_, compared to 272 mAh/g for LiNi_0.8_Mn_0.1_Co_0.1_O_2_, a commonly used intercalation cathode. TMFs are intrinsically difficult to study due to their incompatibility with typical liquid electrolytes and the need for conductive additives to ensure sufficient utilization. In this work, thin-film solid-state devices are used to compare six transition metals mixed with LiF in a 1.1:2 TM/LiF ratio (where TM = Cr, Mn, Fe, Co, Ni, or Cu) without the influence of additive and electrolyte interactions. C/10 delithiated capacities of 540, 113, 402, 407, 566, and 143 mAh/g are shown for (Cr, Mn, Fe, Co, Ni, Cu)-LiF cathodes, respectively. Chromium fluoride consistently outperforms the other cathodes up to 8C (190/219 mAh/g, lithiated/delithiated). Differences between the behavior of the TM-LiF cathodes are explored using electrochemical characterization and atomistic simulations. The choice of TM has a significant impact on cathode performance, which is likely to be connected to their distinct chemical natures, changing the thermodynamics and pathway of the conversion reaction.

## Graphical abstract

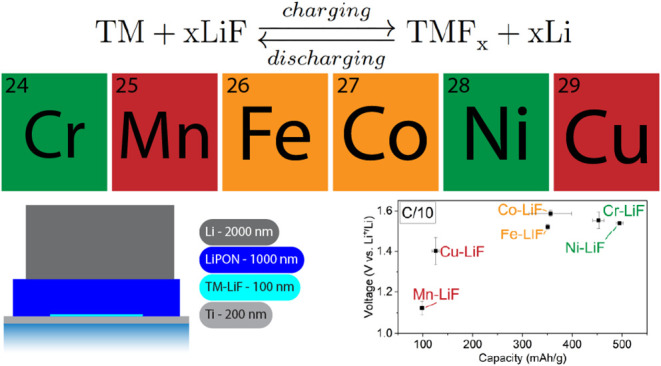

## Introduction

Transition metal fluoride (TMF) conversion cathodes promise up to 4 times higher specific capacities than conventional intercalation cathodes, while still providing reasonably high voltages (2.5 to 3.5 V).^1–3^ Equation 1 shows the general chemical reaction that TMF conversion cathodes undergo during lithiation (forward) and delithiation (backward).

1 $${\mathrm{TMF}}_{x}+x\mathrm{Li}⇌\mathrm{TM}+x\mathrm{LiF}$$

The strong electronegativity of fluorine ions is responsible for the high theoretical electromotive force (EMF) that gives TMFs their promising characteristics as cathode active materials. TMs such as Cr, Mn, Fe, Co, Ni, and Cu are chosen to host the fluorine ions in the delithiated state owing to their redox activity, allowing multiple stable oxidation states. They are also among the lightest and most abundant TMs, both favorable in the context of cathode cost and gravimetric performance.^4^ Figure 1 compares the theoretical voltage and capacity of TMF cathodes to some common intercalation cathodes. TMFs offer potential up to 2.51 Wh/g (CoF_3_). In comparison, two common intercalation-type cathodes, LiNi_0.8_Mn_0.1_Co_0.1_O_2_ (NMC811) and LiFePO_4_ (LFP), can provide theoretical energies of 1.28 and 0.586 Wh/g, respectively.^5,6^ It is important to note that the literature typically reports TMF cathodes based on their delithiated weights, which can inflate performance values by 13–16% by not accounting for the mass of Li in the cathode.^3^

**Figure 1:**
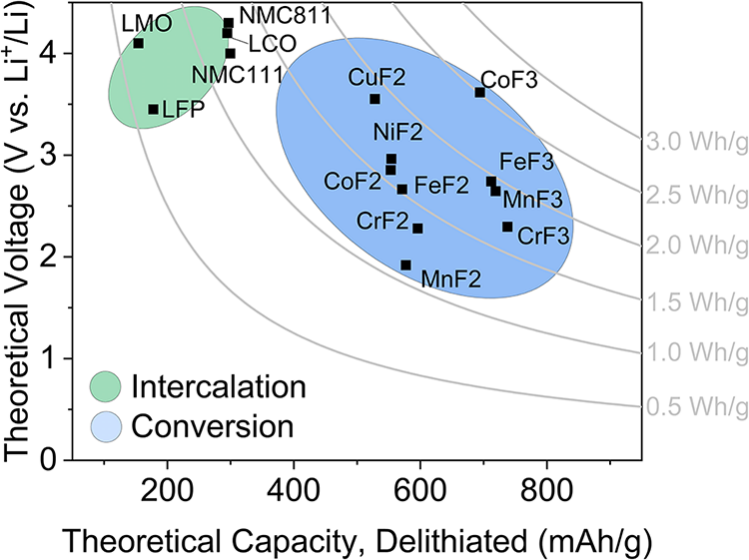
Comparison of theoretical voltage and capacity of TMF conversion and common intercalation cathodes. Theoretical voltages of conversion cathodes were calculated from free energies of formation taken from Thermochemical Data of Pure Substances (Barin I., Platzki G., 1995), and capacities were calculated from formula molecular weights of the delithiated cathodes.^7^ Theoretical values for intercalation cathodes were taken from Julien et al. and Savina et al.^5,6^

Among the TMs studied in this work (Cr, Mn, Fe, Co, Ni, and Cu), all but CrF_*x*_ have been previously reported. This may be due to the reputation of chromium being toxic, making it undesirable to handle. However, a distinction needs to be made between its two most stable oxidation states, Cr­(VI) and Cr­(III). In its hexavalent state, chromium is highly toxic and a proven carcinogen and should be avoided.^8^ In the context of this work, there is some dispute in the literature on whether or not CrF_6_ is stable and/or can be easily synthesized.^9^ In its most stable trivalent state, chromium is biologically essential.^8^ It is not toxic and does not carry the same risks as Cr­(VI). In this work, we attempt to form CrF_2_ and/or CrF_3_. We estimate that overcharging to >10 V may lead to CrF_6_ and/or F_2_ release, which are both unwanted for safety reasons. Figure 1 shows that CrF_2_ (2.28 V, 516/596 mAh/g lithiated/delithiated) and CrF_3_ (2.29 V, 619/738 mAh/g lithiated/delithiated) can compete with other widely used TMFs, at least in theory.

In the literature so far, a systematic comparison of TMF conversion cathodes is not present, and typically, only individual formulations are tested. For Mn, Rui et al. reported an MnF_2_ anode with 300 mAh/g at C/10, which is used as an anode material due to its discharge voltage plateau at roughly 0.5 V.^10^ In addition, this material’s capacity changes over many cycles, likely due to a nanophase restructuring over 10,000 cycles. In Figure 1, MnF_*x*_ shows potential to achieve up to 2.65 V at 719 mAh/g (for *x* = 3). If this is achievable, then this material may still be of interest as a cathode.

Iron-based fluorides are well studied, since iron is cheap, very abundant, and shows high Li^+^ storage capacities at a high voltage (Figure 1).^3,11,12^ Xiao et al. and Zhao et al. report cathode-level (active material only) capacities of 570 (nanocrystal composite, delithiated) and 350 mAh/g (thin-film, equivalent to 402 mAh/g delithiated).^13,14^ These studies manufacture their cells with liquid electrolytes and report compatibility issues such as active material dissolution and fusing, as well as electrolyte decomposition, leading to capacity fading.^3^ Stable iron fluoride cycling has been shown in thin-film solid-state devices in our previous work.^15^ In such a device, iron fluoride could achieve 2000 cycles at 8C and showed 480 mAh/g (C/3.6, lithiated) after 2000 cycles (497 mAh/g theoretical lithiated capacity). Cathode restructuring during cycling is also of high importance for FeF_*x*_ cathodes.^15^

Behl et al. report conversion of Co-LiF (lithiated) cathodes in Li-ion batteries.^16^ They report reversible cycling of CoF_2_ but not CoF_3_ due to the instability of their electrolyte above 4.5 V. Tan et al. report the use of the same material (CoF_2_) as an anode material due to its higher capacity (166 mAh/g) when cycled in a reduced voltage window of 3.2 to 0.01 V.^17^

Villa et al. show a NiF_2_ cathode with 445 mAh/g capacity (delithiated) in the first cycle.^18^ The NiF_2_ cathode exhibited a rapid capacity fade from 445 to less than 50 mAh/g in 6 cycles at C/10. They claim that this is due to their material converting to the insulating LiF phase and then no longer cycling back to the Ni–F phase.

Omenya et al. demonstrate a CuF_2_ cathode with an initial capacity of 450 mAh/g (delithiated) with a capacity fade to 100 mAh/g in 3 cycles.^19^ This was argued to be due to Cu dissolution below the thermodynamic potential of the conversion reaction, likely leading to some extra capacity, as well, albeit not very reversible.

The scientific community is also placing a large effort on optimizing the state-of-the-art for TMFs. For example, electrolyte engineering to help stabilize the cathode–electrolyte interface has helped improve long-term cycling performance.^20^ Other strategies often tackle conversion challenges of cathodes through catalysis. Wu et al. use Ni and Fe together to catalyze and stabilize the required LiF splitting.^21^ In another work, Wei et al. utilize spinel oxides as a redox host for the LiF splitting.^22^ In this way, the energy efficiency of the LiF conversion reaction can be improved by reducing the overpotentials. Despite these advancements, there is limited systematic work on comparing and understanding the performance of pure TMF conversion cathodes.

In this work, we compare a series of six TM cathodes in identical thin-film solid-state devices. Using thin-film deposition techniques and the thin-film cell architecture, we are able to study fundamental active material properties without the need for any additives to avoid unwanted interactions. Furthermore, to avoid dissolution and particle-fusing issues with liquid electrolyte-TMF systems, we employ a solid-state electrolyte (LiPON), which has been shown to have a suitable voltage stability window (up to ∼6 V) and found to be compatible with TMF cathodes in our previous work.^15^ We further support the experimental work with atomistic simulations of structure and ion diffusion driven by machine-learning interatomic potentials (ML-IP) to better understand the experimentally observed trends.

## Results

Figure 2(a) shows a schematic of the coevaporation process used to fabricate the lithiated TMF conversion cathodes. By leveraging the coevaporation technique, we are able to deposit a multiphase cathode that is finely mixed, with phase clusters in the order of a few nanometers in size (Figure 2(b), a high-angle annular dark-field scanning transmission electron microscopy (HAADF-STEM) micrograph).^15^ Figure S1 shows representative scanning transmission electron microscopy-energy-dispersive X-ray (STEM-EDX) elemental maps for an Fe-LiF as-deposited cathode, showing the evenly mixed cathode layer that is obtained through coevaporation. By controlling the deposition rates (calibrated independently) of both phases, we can finely tune the stoichiometry of the TM-LiF thin films. In this work, cathodes were grown with a TM/LiF ratio of 1.1:2. The over-stoichiometry of the metal phase is to ensure that, regardless of minor deposition variations, there is enough metal to accept all of the fluorine ions when fully delithiated. The stoichiometric ratio obtained was confirmed post-deposition using elastic recoil detection analysis (ERDA) depth profiling for Cu-LiF (Figure S2). The measured Cu/LiF ratio is 1.04 ± 0.08:2 for the Cu-LiF cathode. An assumption was made for the work that all cathodes have a TMF_2_ phase that is accessible, which was confirmed with a known crystal phase database search.^23^ We do not limit our cathodes to only accessing the TM­(II) oxidation state, but we do limit the layers to having a maximum known capacity. The capacity of the cathode is controlled by fixing the moles of LiF (7.62 × 10^–7^ mol_LiF_/cm^2^, 73.5 mC/cm^2^, 0.0204 mAh/cm^2^) in the cathode to be the same for all of the metals tested. This means the final layers vary in their thickness (<20% variation) since the volume of metal in the phase is different due to different densities, but all have the same theoretical absolute capacity (1.02 μAh per cell).

**Figure 2:**
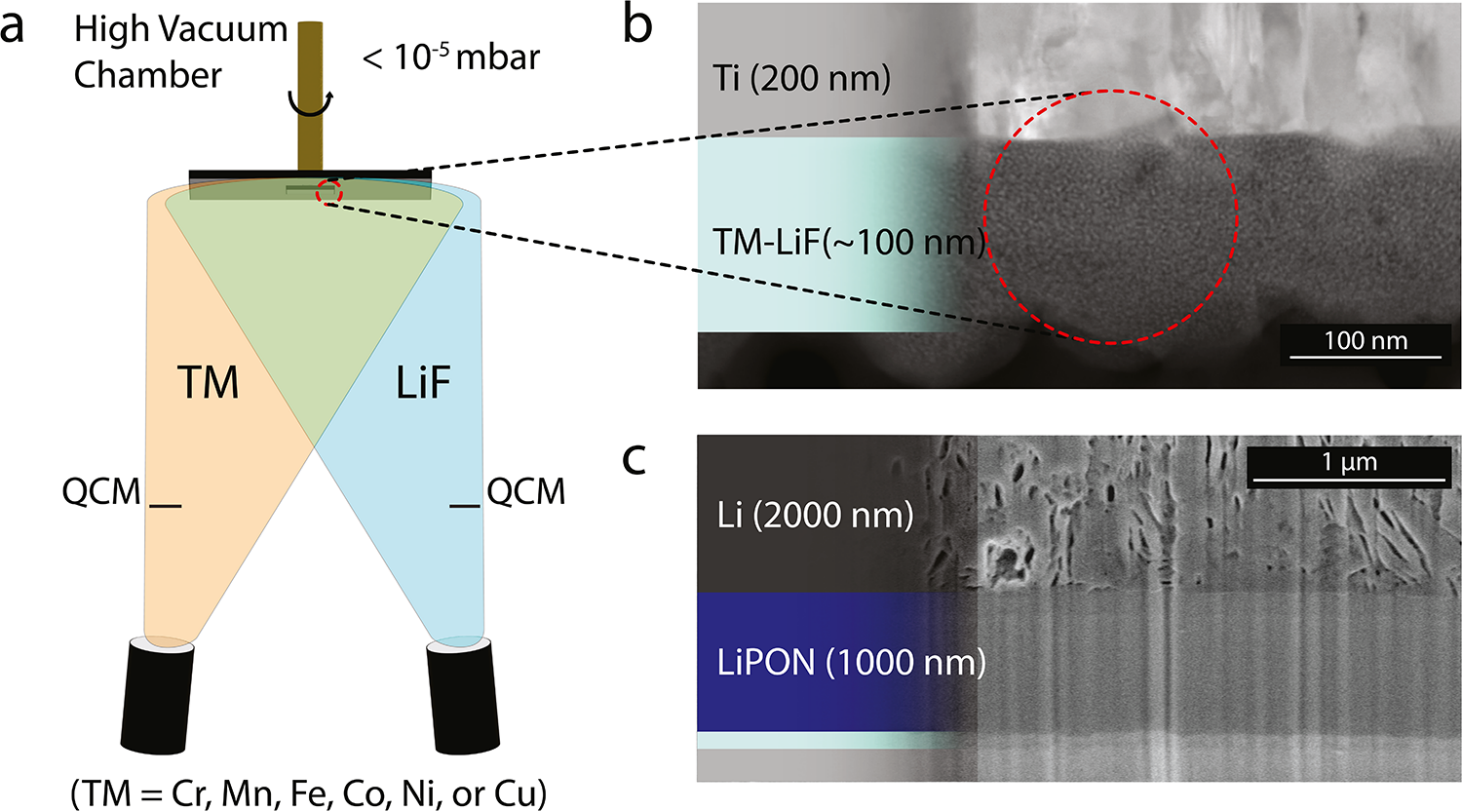
TM-LiF cathode and solid-state cell cross-section. (a) Schematic of the coevaporation step conducted for the fabrication of TM-LiF cathodes. (b) HAADF-STEM micrograph of the as-deposited Fe-LiF cathode (pristine) on the Ti (200 nm) coated substrate. (c) FIB-SEM image of a cell cross-section. Final cell is made up of Glass (1.1 mm), TiN (50 nm), Ti (200 nm), a TM-LiF cathode (90–110 nm), LiPON electrolyte (1000 nm), and Li-metal anode (2000 nm).

Figure 2(c) shows a cross-sectional micrograph of the cell structure. The roughly 100 nm thick cathodes are deposited on a 200 nm thick Ti current collector (as shown in Figure 2(b)). The following layers are 1000 nm of LiPON solid electrolyte and 2000 nm of Li metal for the anode and current collector. The thickness of LiPON was set to 1000 nm to improve the cell success rate (thinner increases the risk of short circuits). In addition, 2000 nm of Li metal was deposited for the anode to ensure there was a large excess of Li in the cell (we are only interested in cathode performance), and to provide a stable reference potential.

In Figure 3, the first three cycles at C/10 (2 μA/cm^2^, 44–48 mA/g) are shown for six different lithiated TMF cathodes. All cells were cycled in the same voltage range (0.5–5 V). Experiments were conducted in a way that all variables are controlled for and the same, except for the metal choice. The upper cutoff voltage was selected to be 5 V in order to maximize cathode activity while avoiding electrolyte degradation. Moreover, a 0.5 V cutoff voltage was selected to avoid lithium metal plating on the cathode. For all cathodes, the first cycle shows a higher capacity than the subsequent cycles. This is reported to be due to the overlithiation of the sputtered LiPON electrolyte and minor self-limiting side reactions.^24^ Apart from the Mn-based cathode, only small variations are present between the second and third cycles of each cathode. Calculated d*Q*/d*V* vs V curves for this experiment (cycle no. 3) are shown in Figure S3.

**Figure 3:**
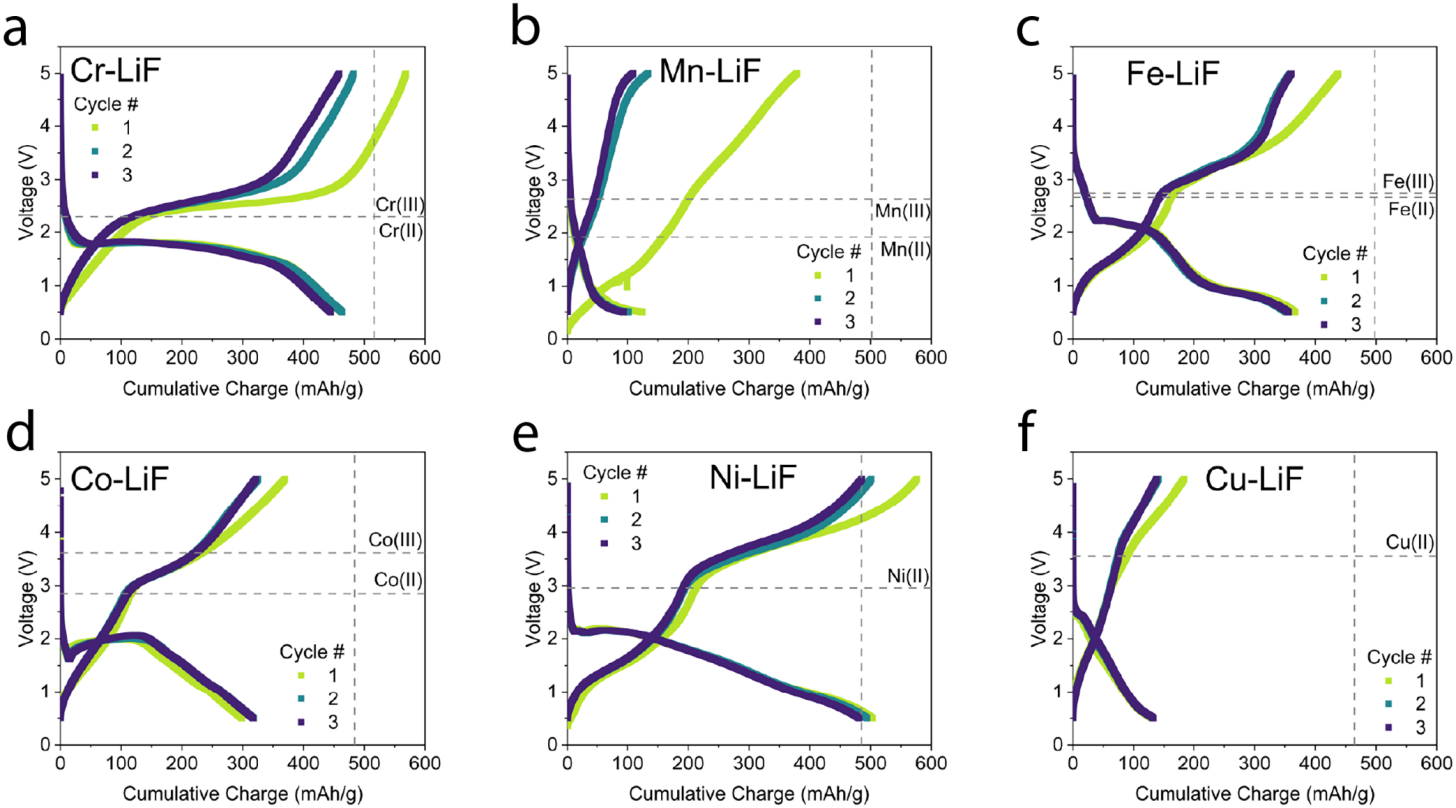
Electrochemical characterization. (a–f) Charge–discharge curves of the first three cycles of each TM-LiF cathode. The cycling rate is 2 μA/cm^2^ (C/10, 44–48 mA/g). Horizontal dashed lines in (a–f) show theoretical bulk EMF values for different formation free energies of the respective metal fluorides (e.g., Cr­(II) is for Cr + 2LiF → CrF_2_ + 2 Li).^7^ Vertical dashed lines represent the theoretical capacity that the cells can achieve (equivalent to fully converting to their TMF_2_ phase). Cumulative charge values are shown for the lithiated cathode. All voltages are versus Li^+^/Li.

Figure 3(a) shows the cycling behavior of the Cr-based cathode. During charge, a single high-capacity plateau is observed between 2.5 and 3 V. During discharge, the majority of the capacity is obtained between 2 and 1.5 V. The graph also shows the representative voltages of the Cr(0) to Cr­(II) and Cr­(III) oxidation (2.28 and 2.29 V, respectively).^7^ It is in this case unclear which oxidation state is being reached by the cell, but previous work suggests that Cr­(III) is the more stable oxidation state.^8^ Figure S3­(a) shows the associated d*Q*/d*V* vs *V* plot as calculated from Figure 3(a). The d*Q*/d*V* vs *V* graph (Figure S3­(a)) shows the presence of a single-step reaction during charge and a two-step reaction during discharge (lithiation of the cathode).

Figure 3(b) shows the results for the Mn-LiF cathode. Compared with all other cathodes in this study, Mn-LiF performs poorly as a conversion cathode. An initial, high-capacity charge half-cycle indicates the conversion of some of the cathode layer to its manganese fluoride (MnF_*x*_) state. The subsequent discharge and subsequent cycles indicate very little reversible capacity in the given voltage range. A discharge plateau starts at roughly 0.6 V, although it is of low importance (for a cathode material) due to its low energy, which is in line with the work of Rui et al. using MnF_2_ as an anode material.^10^ The charge plateau observed at 5 V is also of low importance, especially if it is correlated to the discharge plateau observed at 0.5 V, leading to a low voltaic efficiency. The observed voltage profiles suggest some difficulty in relithiating the MnF_*x*_ phase, indicating a highly asymmetric and/or kinetically limited reaction pathway.^16^

Figure 3(c) demonstrates the cycling behavior of the Fe-LiF cathode. The observed charge and discharge characteristics follow what has been shown in the literature.^13,25,26^ On discharge, an initial intercalative step occurs in the range of 3.5–2.2 V, leading to ∼40 mAh/g of capacity. The related d*Q*/dV vs *V* curve (Figure S3­(c)) indicates a two-step charge and two-step discharge conversion reaction pathway leading to over 350 mAh/g of capacity (lithiated) at C/10.

The Co-LiF cathode cycles are shown in Figure 3(d). As for all of the cathodes, a clear asymmetry is apparent between charge and discharge curves. A pronounced voltage “bounce-back” is observed at 1.6 V with a subsequent voltage increase up to 2 V after 150 mAh/g of capacity. This effect is reported previously and has been attributed to a self-activating mechanism, where a small degree of lithiation of the cathode improves kinetic attributes of the cathode, leading to lowered overpotentials for the subsequent cathode lithiation.^12,27^ As the discharge rate is already slow (C/10), this observation indicates poor kinetic performance of the cathode material. The total specific capacity is also lower than that for Cr, Fe, and Ni. Considering the d*Q*/d*V* vs *V* plot of Co-LiF (figure S3­(d)), it is accessing only the Co­(II) oxidation state to make CoF_2_. Conversion to CoF_3_ likely requires voltages above 5 V, based on this experiment, indicating significant overpotentials for the reaction.

Figure 3(e) shows the charge–discharge curves for the Ni-LiF cathode. Similarly to the Co-system, the Ni-based cathode also shows a “bounce-back” effect and charge–discharge asymmetry. Regardless, with the experimental conditions used here, Ni-LiF shows the highest specific capacity of 495 mAh/g (or 566 mAh/g delithiated). Notably, the capacity is 2% higher than the theoretically achievable capacity for NiF_2_ of 554 mAh/g. This discrepancy can come from small errors in deposition conditions and/or additional capacity from an interfacial storage mechanism at low voltage.^28^

Finally, Figure 3(f) shows the Cu-LiF cathode with a specific capacity of 120 mAh/g, which is low in comparison to its theoretical value of 480 mAh/g and in comparison to the other cathodes tested. Unlike the Mn-LiF cathode (Figure 3(b)), Cu-LiF does not show any significantly higher capacity during its first charge and shows better voltaic efficiency. This would suggest that the factors limiting the performance of the Mn- and Cu- cathodes are different. As argued above, considering the first 3 cycles of Mn-LiF, the first charge has more than half of the theoretical capacity of the cell. This suggests that the cathode was somewhat delithiated, forming an MnF_*x*_ phase. In contrast, Cu-LiF shows a very small first charge capacity, indicating that the reason for the inactivity of the Cu-LiF cathode is very different in nature from that of the Mn-LiF. As will be discussed later, CuF_2_ is less stable in comparison to the other TMFs considered in this work (Figure 6). This implies that the delithiation of Cu and LiF (formation of CuF_2_) is less favored than with other TMs, where TMF_*x*_ is more stable.

The results of faster cycling (C-rate test) are shown in Figure 4. The theoretical cycling rates range from C/2 to 8C (10 to 160 μA/cm^2^, or ∼0.23 to ∼3.68 A/g). The cycling protocol was set to consist of 10 cycles at C/2, 1C, 2C, 4C, and 8C, and again at 1C for a total of 60 cycles. The shaded regions highlight the two sets of 1C cycling.

**Figure 4:**
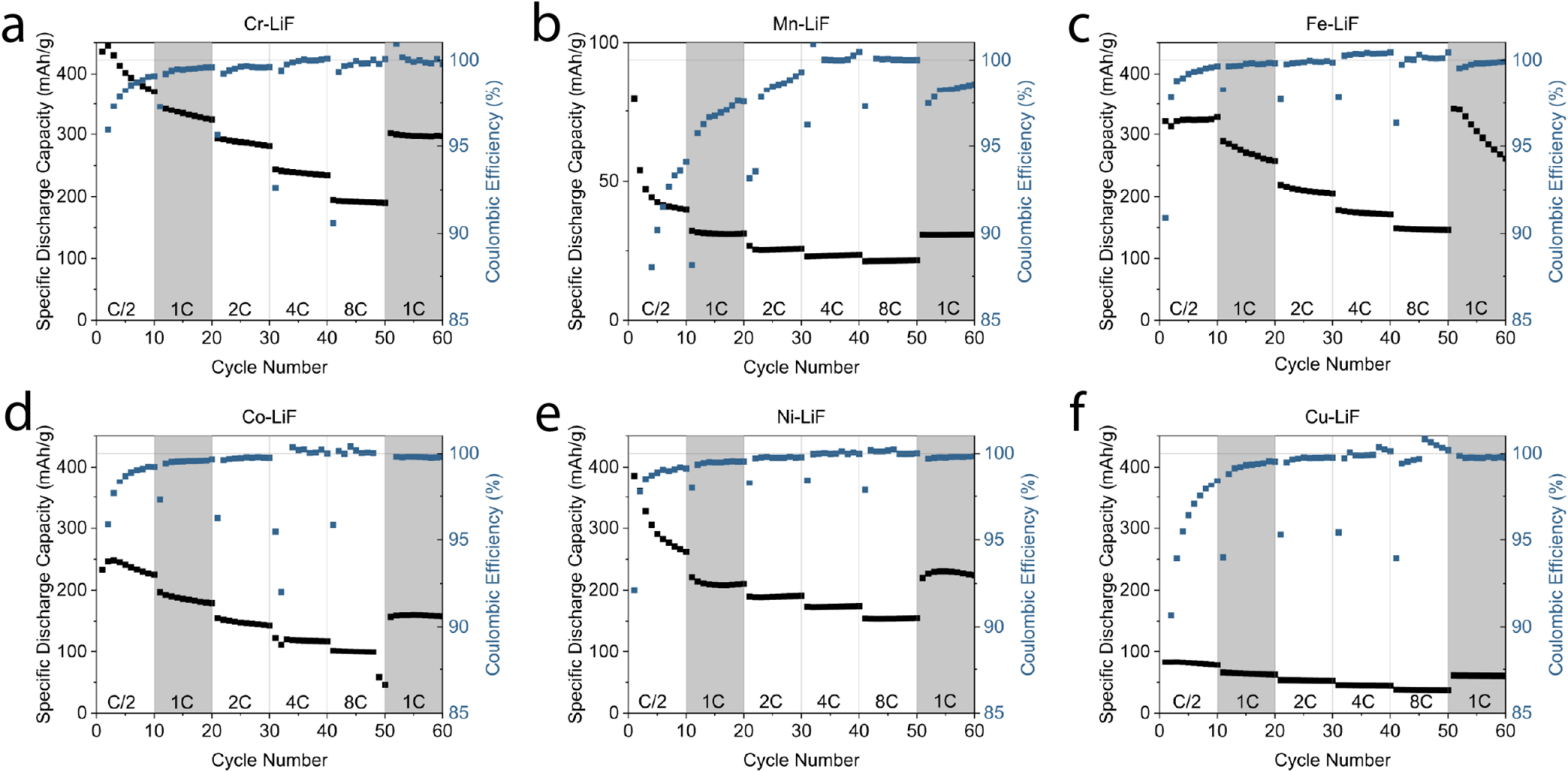
Rate test cycling. (a–f) Specific discharge capacity (left axis, black) and respective Coulombic efficiency (right axis, blue) of TM-LiF cell over 60 cycles at current densities of 10 to 160 μA/cm^2^ (C/2 to 8C assuming 20 μAh/cm^2^).

Figure 4(a) shows the rate-dependent cycling for Cr-LiF. Its capacity declines quickly at C/2 from 436 to 370 mAh/g in the first 10 cycles. At 8C (cycles 41 to 50), the cathode still shows 190 mAh/g of discharge capacity. The degradation rate (slope of the black lines) is also reduced at higher cycling rates, although this is likely due to the decreased cathode utilization at high rates. If the degradation mechanism is dependent on cathode utilization, the degradation per cycle will decrease at higher rates. Cathode capacity is stable in cycles 51 to 60 (1C) at 297 mAh/g.

Mn-LiF rate performance is shown in Figure 4(b). Note the change in the *y*-axis scale. This cathode is again of low interest due to its poor capacity and Coulombic efficiencies for the reason suggested previously.

The cycling behavior of Fe-LiF is shown in Figure 4(c). The capacity of this cathode ranges from 350 (C/10) to 146 mAh/g (8C). As shown in our previous work, the Fe system benefits from fast cycling to improve its nanostructure, ultimately improving some kinetic aspects, leading to better cathode utilization.^15^ This is visible in Figure 4(c) through the increase of capacity observed in the shaded regions (at 1C), after 30 cycles at higher rates. This preferential nanostructure is quickly changed back when returning to the slower cycling, leading to the 23% drop in capacity during the last 10 cycles at 1C. This capacity is recoverable with high-rate cycling.^15,29^

Co-LiF cycling is shown in Figure 4(d). The cathode capacity ranges from 248 (C/2) to 100 (8C) mAh/g. From cycles 51 to 60 at 1C, Co-LiF capacity shows stable cycling with 160 mAh/g of discharge capacity. This is roughly half of the capacity measured at C/10, indicating significant rate-dependent overpotentials, typical of conversion cathodes.

Ni-LiF (Figure 4(e)) is another promising conversion cathode. Despite a rapid capacity loss in the first 5 cycles from 386 to 280 mAh/g at C/2, the cathode stabilizes at a practical discharge capacity of 226 mAh/g at 1C (cycles 51–60). Furthermore, Ni-LiF achieves 154 mAh/g at 8C (second highest, after Cr-LiF).

Figure 4(f) shows the poor performance of the Cu-LiF cathode. The maximum capacity reached is 82 mAh/g at a rate of C/2. At 8C, Cu-LiF shows a discharge capacity of 37 mAh/g. These low values are likely due to some overpotentials hindering the conversion mechanism from taking place.

## Discussion

Figure 5(a) summarizes the average discharge voltage and specific discharge capacity of the 6 cathodes, which are obtained from the data shown in Figure 3. With the exception of Mn-LiF and Cu-LiF, the average discharge voltages obtained are similar. It is worth noting that the measured capacities for Ni- and Cr- cathodes are the closest to theoretical values for NiF_2_ (500 vs 485 mAh/g, lithiated) and CrF_2_ (452 vs 516 mAh/g, lithiated). As previously mentioned, Ni-LiF likely shows inflated capacities due to an interfacial storage mechanism and/or a small error in deposition.^28^ The voltages of all cathodes are, however, significantly lower than those expected thermodynamically. This is typical and was observed previously as a major drawback for conversion systems. The reason for this discrepancy is most often attributed to sluggish kinetics leading to high overpotentials.^3,30^

**Figure 5:**
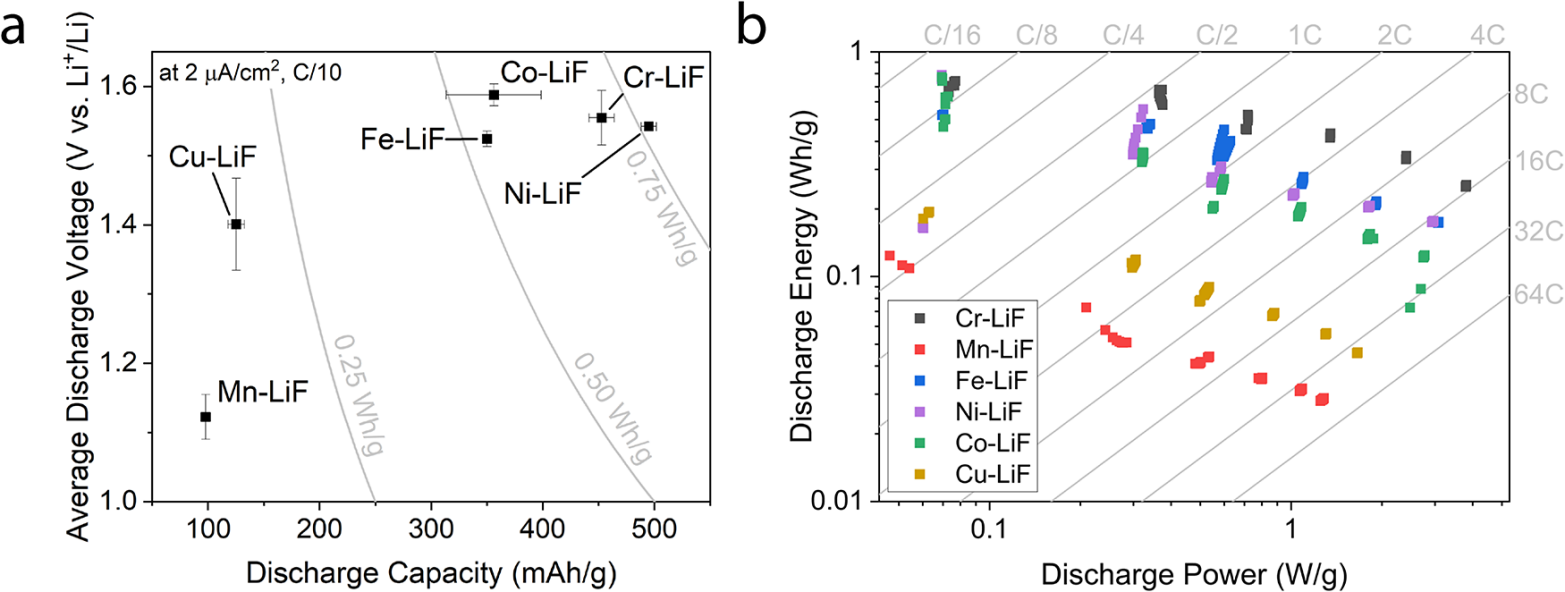
Cathode performance. (a) A comparison of specific discharge capacity and average discharge voltages (*V* vs Li^+^/Li) of TM-LiF conversion cathodes at 2 μA/cm^2^ (theoretical charging rate of C/10). The error bars represent 1 standard deviation for each cathode for a minimum of 4 cells each (three C/10 cycles each). The gray lines indicate the cathode-level specific energy. (b) A Ragone plot comparing discharge specific power and discharge specific energy of the 6 different cathodes. The gray lines and labels indicate the actual charging rates of the cathodes. All values are specified in the lithiated state.

Figure 5(b) shows a Ragone plot for all six tested TMF cathodes. The highest cathode discharge energies of 0.791 Wh/g (all values specified at the lithiated cathode level) are observed for Ni-LiF at the lowest tested power of 69.3 W/kg. At the highest tested power densities (theoretical 8C, actual >16C), Cr-LiF shows the highest discharge energy of 0.256 Wh/g at 3.81 W/g. Cr-LiF shows the highest discharge energy consistently at all tested powers above 0.100 W/g. The underlying reasons for the stark differences in measured performance are explored further.

Three potential hypotheses were initially considered to explain the differences in the TMF_*x*_ cathode performance:

1. Thermodynamic (bulk) material properties of the lithiated or delithiated materials.
2. Charge (Li^+^ and/or e^–^) mobility through the active material.
3. Chemical reaction pathways and overpotentials of lithiation/delithiation reactions.

### Hypothesis 1

Theoretical TMF reaction voltages (Figure 2) and the enthalpies of formation of the various considered phases (Figure S4) can provide some insight into the thermodynamic differences between the TMs and their corresponding fluoride phases. Based on the enthalpies of formation of the relevant binary and ternary phases (Figure S4), no reason for a Cr and Ni advantage is observed. The calculated values consider bulk material properties only and negate the entropic effects. Previous reports explain the importance of considering nanoscale contributions in the context of thermodynamics. Van der Ven and Wagemaker suggest that the electrode potential (Gibbs free energies of lithiation) can strongly depend on particle size.^31^ Such a contribution can make electrodes show more solid-solution-like voltage profiles. This could explain the sloping nature of the voltage curves in Figure 3 as well as the low average discharge voltage compared to theory. Nevertheless, we expect a similar starting nanostructure for all cathodes, as they were manufactured in the same way.

Alternatively, we notice a curious correlation between the experimentally measured cathode capacity and the melting point of the corresponding TMF, as visible in Figure 6(a). Generally, the melting point of an inorganic compound reflects the temperature at which the thermal energy is sufficient to break the crystal lattice so that the solid becomes liquid, and it is influenced by numerous parameters such as bond strength, lattice energy, crystal structure, oxidation state, presence of defects and impurities, etc. Melting points of fluorides are relatively high, from 1100 to 1800 K, due to the strong metal–fluorine bonds, whereas MnF_2_ and CuF_2_ have the lowest melting points of the considered fluorides. Another interesting observation depicted in Figure 6(b) is that TMFs with high melting points exhibit higher capacities, especially considering the extreme cases of Cu (low melting point, low capacity) and Ni (high melting point, high capacity). Despite the observed trend, using bulk melting points to determine the performance of TMFs is not a valid strategy. Notably, CrF_2_ does not match the trend. More likely is that many interconnected phenomena play a role and that some of those also manifest in the bulk melting points observed.

**Figure 6:**
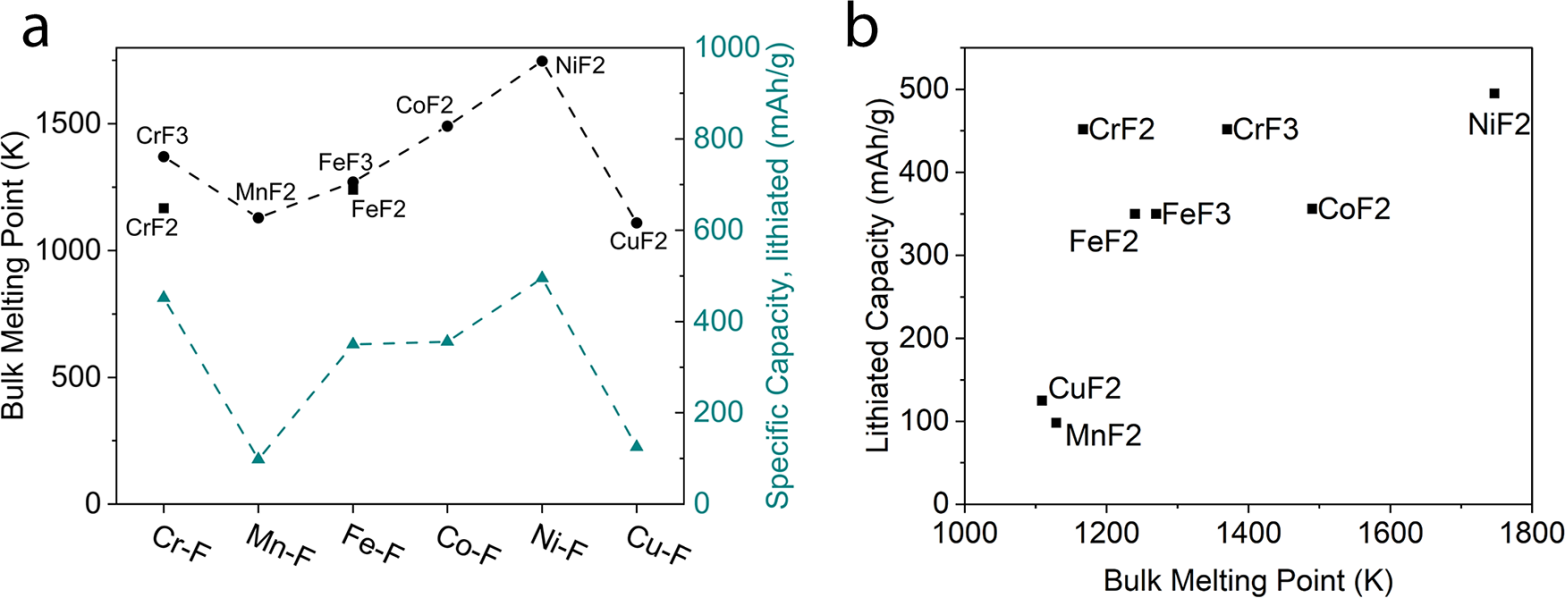
Thermochemical trends. (a) Bulk melting point (*K*, left axis) and lithiated capacity (mAh/g, right axis) for di- and trifluorides of the 6 TMs. Dashed lines serve only to compare trends. (b) Lithiated capacity vs bulk melting point of TMFs studied in this work.

It is unclear why the observed discharge capacity correlates with the melting point. One can speculate that the greater stability of the TMF lattice, manifested in their high melting point (such as for the fluorides of Ni, Co, and Cr), makes the charging (delithiation) process more favorable as compared to TMFs with lower lattice stability and lower melting point (such as the fluorides of Cu and Mn). As the formation of the TMF phase upon charging is often considered to limit the performance of these cathodes,^3^ the favorable formation of TMF crystallites should facilitate the delithiation, reducing inherent overpotentials and improving the observed capacity. This phenomenon would presumably have the opposite effect upon lithiation (discharging) due to the higher lattice stability of the TMF that exhibits good performance. It is known that the lithiation step is usually facilitated by the initial lithiation due to the formation of more conductive phases; we still believe this explanation holds.^3^ It is worth noting that opposite trends are known for conventional intercalation-type cathodes. For instance, Li­[Ni_*x*_Mn_*y*_Co_*z*_]­O_2_ (NMC) cathodes with higher thermal stability (lower thermal decomposition point) exhibit lower discharge capacities, albeit higher capacity retention.^32^

### Hypothesis 2

To complement the conductivity measurements and to gain atomic-scale insights into ion transport behavior, we used advanced materials modeling techniques. In particular, to investigate Li-ion diffusion for long time scales (ns) that are inaccessible using ab initio-based techniques, we performed molecular dynamics (MD) simulations for all six TMF rutile phases using machine learned interatomic potentials (MLIPs), which have not been widely applied to examine ion diffusion in such conversion cathodes. While maintaining quantum mechanical accuracy at reduced computational cost, the MD simulations were run for the six TMFs (doped with 8% Li, 780 atom structures) at 500 K and for a time scale of 1.5 ns (which is an order of magnitude greater than current ab initio MD), and illustrated in Figure 7, showing the Li-ion diffusion pathways.

**Figure 7:**
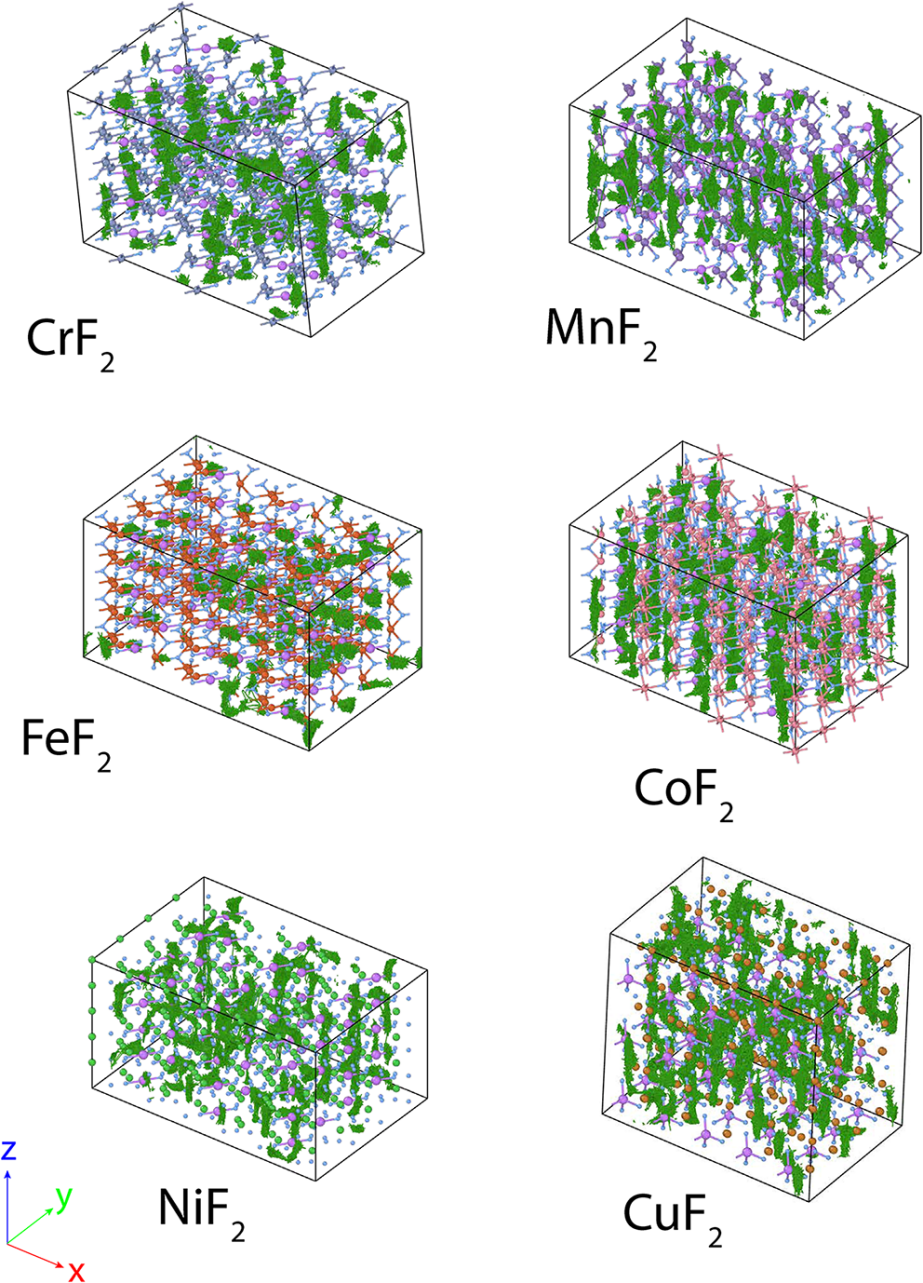
Computed Li trajectories. MD-simulated structures of rutile TMF_2_ with 8% Li doping showing the Li-ion trajectories and diffusion pathways (green lines). All structures are made up of 780 atoms and show trajectories for 1 ns (at 500 K).

An important factor for understanding the lithium transport flux through the TMF structures is the Li-ion diffusion rate quantified by the diffusion coefficient (*D*_Li_) Figure 8(b), which was derived from the temperature-dependent mean-square displacements (MSDs) shown in Figure 8(a). Direct comparison with experimental *D*_I_ values can be difficult, as they are not straightforward to measure for complex TMFs. Nevertheless, these diffusion coefficients are qualitatively compared with experimental conductivities determined through electrochemical impedance spectroscopy (EIS) measurements and equivalent circuit model fitting (Figure 8(c)). These experimentally determined mixed conductivities of the cathode and LiPON were fitted through EIS (Figure S5) in the charged state (charged to 5 V at C/10, followed by a voltage hold until the current dropped below C/100).

**Figure 8:**
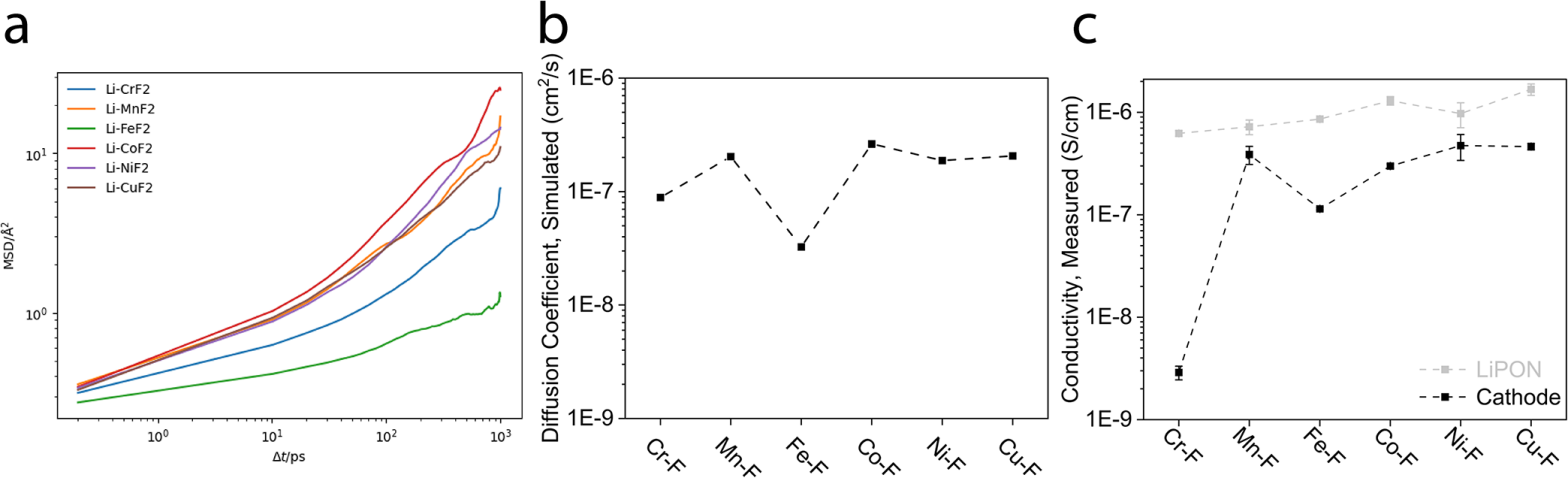
Lithium and electron mobility. (a) Mean squared displacements (MSDs) of Li in the TMF_2_ rutile phases. (b) Computationally determined Li^+^ diffusion through 8% Li-doped TMF_2_ rutile phases. (c) Experimentally determined conductivities of the cathode and the LiPON electrolyte were determined through EIS measurements and equivalent circuit model fitting (Figure S5).

The simulated Li-ion trajectories in the TMF structures (Figure 7) show diffusion pathways through channels along the *z* direction, with the exception of CrF_2_ and FeF_2_ structures, which show no preferential channel migration. The trend is further solidified in Figure 8, where CrF_2_ and FeF_2_ show the lowest diffusion coefficients and the lowest mixed ionic-electronic conductivities. The results, however, suggest that this hypothesis on charge mobility is not supported since the device-level performances (Figure 5) of Cr-LiF and Fe-LiF are not the weakest candidates, and both Mn-LiF and Cu-LiF show high diffusion coefficients and conductivities but perform poorly in devices. Overall, experimentally determined mixed ionic-electronic conductivities and MD-simulated Li-ion diffusion coefficients follow trends similar to each other, but do not fully align with electrochemical performance trends.

The mixed ionic and electronic mobilities shown in Figure 8(b,c) can also be considered in the context of bulk cathodes. Such values can inform particle sizes of active material within the cast bulk composite cathodes. Although it is not in the scope of this work, it would follow that TMFs with poorer conductivities would need smaller particle sizes and a larger fraction of conductive additives in order to achieve sufficient ionic and electronic conductivities within the composite cathodes.

### Hypothesis 3

The dramatic phase change during cycling for TMF conversion cathodes (bulk and otherwise) may play a significant role in device-level performance. Based on this work, we suggest that the reaction pathway for the lithiation or delithiation of the cathode active material plays a major role in controlling its performance. In this way, the chemical nature of the TM selected can affect the cathode reaction pathway and conversion step. In addition, different interfacial effects between LiF and TMs will greatly affect their performance. The stability, concentration, and dimensions of the intracathode interfaces can impact reactions and charge mobility. Liu et al. discuss the overpotentials associated with the nucleation of the Fe-LiF phases during lithiation of their cathode.^33^ This problem seems to be further exacerbated by nanoscale reaction phenomena previously discussed that are relevant in all cathode geometries (bulk, thin-film, single-crystal, etc.). Smaller particles can stabilize more homogeneous lithiation phenomena, which can access solid-solution-like reactions. This can both help and hinder electrode performance.^30^ In this work, we can only argue that the chemical nature of the TM used for the LiF splitting (and reforming) reaction also provides a non-negligible effect. This leads us to stand for the hypothesis that the TM choice can affect the reaction pathway significantly enough to limit its usefulness as a TM for TMF conversion cathodes. Moreover, Figure S6 shows phase diagrams of Li-TM-F for the six metals in question. We observe that for the poor-performing cathodes (specifically Mn-LiF and Cu-LiF), the most direct route for cathode (de)­lithiation does not involve a ternary structure of the type LiTM_*x*_F_*y*_. The absence of these ternary structures likely leads to a single reaction step between TMF_*x*_ and TM + LiF. Such a reaction step may carry higher activation energies as compared to a multistep path, where incremental lithiation is somewhat stabilized. Kinetic and nanoscale contributions may also stabilize other intermediate phases that are not obvious through bulk thermodynamics.^34^ Karki et al. show that the FeF_2_ lithiation is topotactic in nature, leading to a well-defined lithiated structure allowing for percolation of electrons through it. In contrast, Wang et al. describe a coarsening of Cu nanoparticles within CuF_2_ cathodes as they are cycled, leading to a quick capacity fade.^35^ They attribute this to a higher diffusion coefficient of Cu vs Fe ions. Naturally, this hypothesis should be further explored through operando chemical analyses and or simulation work that also takes into account the nanoscale nature of conversion cathodes.

## Conclusions

In this work, the performance of six different transition metal (Cr, Mn, Fe, Co, Ni, and Cu) fluoride conversion cathodes was compared in a thin-film solid-state configuration. It was found that Cr-LiF and Ni-LiF cathodes exhibited the highest specific capacities (452/521 and 495/566 mAh/g, lithiated/delithiated, respectively) at C/10 cycling. On the other extreme, Mn-LiF and Cu-LiF both showed poor reversibility and/or low specific capacities in the first three cycles. When comparing specific energies, Co-LiF was comparable to Cr-LiF and Ni-LiF cathodes due to its slightly higher average discharge voltage (1.59 V). At almost all cycling rates, Cr-LiF outperformed the other cathodes up to the highest tested rate of 8C (190/219 mAh/g, lithiated/delithiated). For the high-performing cathodes, almost full theoretical capacity is achieved. The main challenges that remain are the low discharge voltage and the large voltage hysteresis observed for the TMF cathodes.

We proposed and scrutinized three hypotheses that could explain the differences between the TMF cathodes. Thermodynamic properties such as the bulk TMF melting point and bulk formation energies may explain the resulting trends. We speculate that the bulk melting point and cathode performance are correlated due to underlying phenomena, such as TMF lattice stability, that are shared between the two properties. Experimentally determined mixed ion conductivities and simulated bulk Li diffusion coefficient values followed similar trends to each other, but could not fully elucidate electrochemical trends. Due to this, we argue that although diffusion does affect the performance of TMF cathodes, it is not the distinguishing factor for their performance in the thin-film solid-state configuration. Finally, we suggest that the dramatic phase changes occurring in TMF cathodes during (de)­lithiation are strongly impacted by the choice of TM by changing reaction pathways and/or the cathode nanostructure.

## Experimental Section

### Fabrication

Uncoated Corning Boro-Aluminosilicate glass (CB-0111, Delta Technologies, Ltd., 25 mm × 25 mm × 1.1 mm) was wiped clean with isopropyl alcohol and a lint-free tissue. The bare glass was plasma-etched under Ar gas in a CT200 magnetron sputtering cluster (Alliance Concept) for 20 min. Subsequently, TiN with a thickness of 50 nm was coated using the CT200 at 400 °C by DC magnetron sputtering of a 25 cm diameter target of Ti (gas flow of 120 sccm Ar and 10 sccm N_2_, working pressure of 3 mTorr) with a power of 3.1 W cm^–2^. Ti was then deposited with a thickness of 200 nm using the same tool and target (21 sccm Ar gas flow, 2 W cm^–2^ power, and 3 mTorr pressure). The cathode was deposited to the desired thickness through a custom circular shadow mask (for 5 mm^2^ cells) using a Nextdep thermal evaporator (Angstrom Engineering Inc.) by coevaporating the metals (Cr, Mn, Fe, Co, Ni, or Cu) and lithium fluoride inside alumina crucibles (and a Ni crucible liner for LiF). For more information, please see our previous publication.^15^ The material source specifications are listed in Table 1. The deposition rates of both materials were controlled independently during deposition using multiple quartz-crystal microbalances to achieve a stoichiometric ratio of 1.1:2 metal/LiF. The depositions were designed in such a way that the LiF loading was identical for all cathodes (7.62 × 10^–7^ mol_LiF_/cm^2^, 73.5 mC/cm^2^, 0.0204 mAh/cm^2^). The solid electrolyte, LiPON, was deposited to a thickness of 1000 nm through a custom electrolyte shadow mask using an Orion sputtering system (AJA International Inc.). The LiPON was deposited using RF cosputtering of Li_3_PO_4_ and Li_2_O 2” targets in a confocal arrangement at a gas flow of 50 sccm N_2_, a power of 4.93 and 5.92 W cm^–2^, respectively, and a working pressure of 3 mTorr. The Li metal (99+%, 211442500, Thermo Fischer Scientific Inc.) anode was deposited through a custom anode shadow mask using the same thermal evaporator as described above to a thickness of 2000 nm using a stainless steel crucible arc-coated with alumina.

**Table 1:** List of Materials Used during Thermal Evaporation with Their Respective Purity, Catalog Number, and Supplier

material	purity [%]	catalog number	supplier
chromium (Cr)	99.99	014760.18	Thermo Fisher Scientific Inc.
manganese (Mn)	99.98	036215.14	Thermo Fisher. Scientific Inc.
iron (Fe)	99.95	042383.22	Thermo Fisher Scientific Inc.
cobalt (Co)	99.95	042353.14	Thermo Fisher Scientific Inc.
nickel (Ni)	99.995	042333.18	Thermo Fisher Scientific Inc.
copper (Cu)	99.999	010953.30	Thermo Fisher Scientific Inc.
lithium fluoride (LiF)	99.99	014463.18	Thermo Fischer Scientific Inc.

### Characterization

Electrochemical characterization was performed in an Ar-filled glovebox at room temperature with no applied cell pressure using Squidstat Plus (EIS measurements) and Squidstat Prime (DC measurements) potentiostats (Admiral Instruments). The reported capacities correspond to electrode-level capacities (lithiated or delithiated) calculated using calibrated material volumes and theoretical material densities. Applied currents range from 2 to 160 μA/cm^2^ in potential ranges from 0.5 to 5 V (vs Li^+^/Li). Electrochemical impedance spectroscopy was done using a perturbation amplitude of 50 mV in a frequency range of 2 MHz–0.5 Hz (12 steps per decade) after charging or discharging the cells at C/10 and performing a voltage hold until the current dropped below C/100.

Samples for transmission electron microscopy (TEM) were transferred to a Helios 660 (Thermo Fischer Scientific) FIB-SEM dual beam microscope using an airless transfer module. The TEM lamellae were initially thinned using a 30 kV Ga beam at a range of currents (from 9.3 nA to 80 pA), followed by polishing at 8, 5, and 3 kV with lower ion beam currents (∼60 pA) until satisfactory electron beam transparency was achieved. The thinned lamellae were transferred to an Ar-filled glovebox for storage and then to TEM using an airless transfer holder (Gatan). The TEM experiments were performed on a JEOL JEM-F200 microscope operated at a 200 kV accelerating voltage.

Heavy Ion ToF-ERDA (HI-ERDA) measurements were conducted with a 13 MeV iodine beam under a total scattering angle of 36° at the Laboratory of Ion Beam Physics at ETH Zurich.^36^

## Computational Methods

### Density-Functional Theory

The electronic structure of atomic models in this work was evaluated using density-functional theory (DFT) calculations using version 6.4.1 of the Vienna Ab-initio Simulation Package (VASP).^37–40^ The r^2^SCAN meta-GGA exchange–correlation functional was used due to its accuracy for a wide range of materials, including correlated systems that include transition metal ions.^41^ The calculations were carried out using a plane-wave basis with a kinetic-energy cutoff of 520 eV; given the large size of the evaluated supercells, the Brillouin zone was sampled only at the Γ-point. Gaussian smearing of the electronic states was used with a width of 0.05 eV. The VASP recommended projector-augmented wave (PAW) sets were used for all elements.

The electronic energy is converged down to 1 × 10^–6^ eV during every self-consistent field (SCF) cycle using a preconditioned conjugated gradient algorithm (ALGO = ALL). The structures were optimized to a maximum force difference of 1 × 10^–2^ eV Å^–1^ between ionic steps using a residual minimization method. These settings were used to optimize the geometry and cells of the atomic structures considered in this work.

### Ab Initio Molecular Dynamics

To sample structures, we have employed *ab initio* molecular dynamics (AIMD) as implemented in VASP. We use a time step of 2 fs to integrate the equations of motion. A Langevin thermostat was used to control the temperature, with a coupling constant of 10 ps^–1^, while lattice degrees of freedom were constrained with a friction coefficient of 100 ps^–1^ using the Parrinello–Rahman barostat as implemented in VASP. In this way, we sampled from the isothermal–isobaric ensemble (NpT). Pressure was set to 0 kbar, and temperatures in the range 150–1500 K were explored.

### Fine-Tuning CHGNet

In order to scale our calculations to larger model sizes and longer trajectories needed to evaluate dynamical properties, we made use of an ML-IP. To get an approximate potential-energy surface for our systems, we have fine-tuned the foundational CHGNet graph neural network (GNN) model (version 0.3.0) with DFT samples from our AIMD runs.^42^ To retain the learned knowledge of the foundational model, we have partially frozen layers of the network before retraining. Table S1 shows a list of the DFT runs that generated the structures used for the fine-tuning. The cross-validation plots of the retrained models can be found in Figure S7 in the Supporting Information.

### Evaluating Diffusion Coefficients

We have used the fine-tuned CHGNet model to run classical MD simulations on supercells of 780 atoms of the different TMF_2_ structures, where TM is Cr, Mn, Fe, Co, Ni, and Cu, with 8 atom % (atomic percent) added Li. Classical dynamics is carried out in the NpT ensemble at a temperature of 500 K and 0 kbar pressure. To evaluate the diffusion of Li atoms in the structure, we calculate the mean-square displacement (MSD) ⟨(**x**(*t*) – **x**(0))^2^⟩ and obtain the diffusion coefficient as the slope of the MSD versus time plot at long times.

$$D=\lim_{t\to \infty }\frac{1}{n}\frac{\left\langle \sum_{1}^{n}{\left(x_{i}\left(t\right)-x_{i}\left(0\right)\right)}^{2}\right\rangle }{6t}$$

The kinisi library was used to evaluate a robust Bayesian estimate of the diffusion coefficient from our trajectories.^43^ By comparing the MSD plots for different lengths of trajectories, we found that they tend to converge after about 0.5 ns of dynamics.

### Structure Generation and Visualization

The manipulation of atomic structures was done with in-house scripts that made use of the ASE library. Atomic structures were visualized using ovito.^44^

## Supplementary Material



## Notes

STEM-EDS elemental maps of Fe-LiF; ERDA depth profile of the Cu-LiF cathode; d*Q*/d*V* vs *V* for all 6 cathodes; enthalpies of formation of different fluoride phases of each of the 6 cathodes; impedance spectra (EIS) of all 6 cathodes in charged and discharged states; a list of structures used for DFT computations and for machine-learning fine-tuning; predicted Li-Me-F phase diagrams for all 6 metals; benchmarking of fine-tuned CHGNet models for all 6 metals; necessary References (PDF)
