# Supplementary material for: Performance Comparison of Transition Metal (Cr, Mn, Fe, Co, Ni, Cu)-Fluoride Conversion Cathodes in Thin-Film Solid-State Batteries
Source: ACS Appl Energy Mater. 2025 Oct 3;8(20):15101–11. doi: 10.1021/acsaem.5c01772 (PMC12569967; doi:10.1021/acsaem.5c01772)
Supplement: Supplementary file 1 [file ae5c01772_si_001.pdf]

Supporting Information - Performance Comparison of Transition Metal (Cr, Mn, Fe, Co, Ni, Cu)-Fluoride Conversion Cathodes in Thin-Film Solid-State Batteries

Joel Casella,*,† Jędrzej Morzy,† Felix C. Mocanu,‡ Arnold Müller,¶ Maksym Yarema,§ Moritz H. Futscher,† M. Saiful Islam,‡ and Yaroslav E. Romanyuk*,†

†*Laboratory for Thin Films and Photovoltaics, Empa - Swiss Federal Laboratories for Material Science and Technology, Dübendorf, 8600, Switzerland*

‡*Energy Materials Research Group, Department of Materials, University of Oxford, Oxford, OX1 3PH, United Kingdom*

¶*Laboratory of Ion Beam Physics, ETH Zürich, Zürich, 8093, Switzerland*

§*Institute for Electronics, Department of Information Technology and Electrical Engineering, ETH, Zurich, 8092, Switzerland*

E-mail: joel.casella@empa.ch; yaroslav.romanyuk@empa.ch

Supporting Information Available

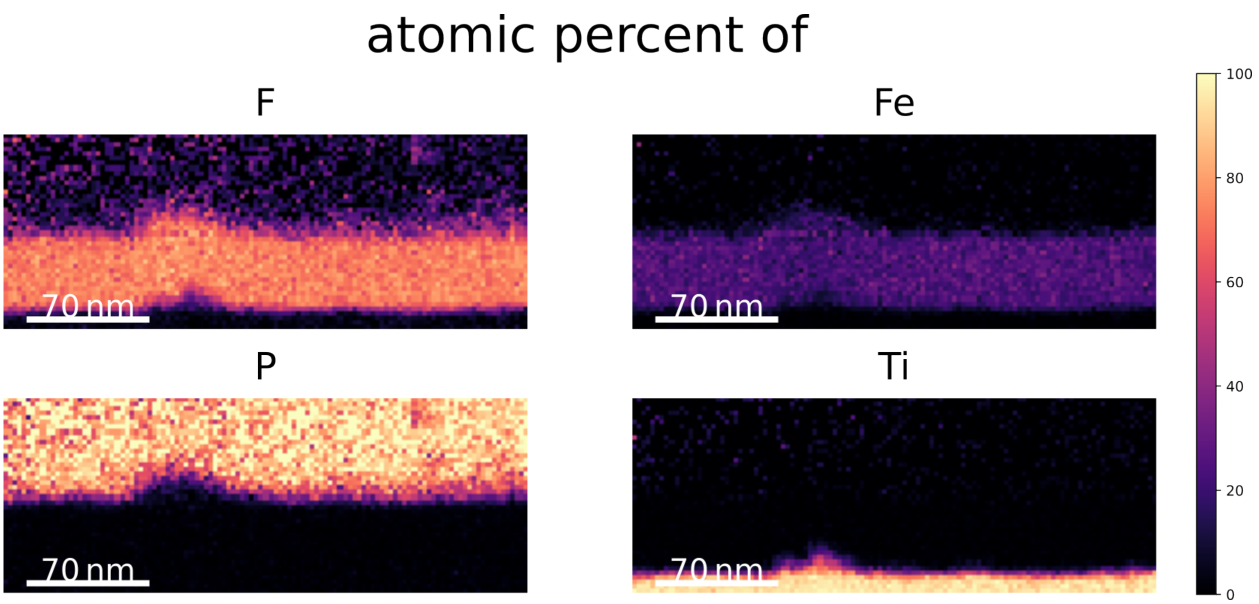

Figure S1: Representative EDX spectra for a Fe-LiF cathode showing the atomic percent of F, Fe, P and Ti. This particular sample is a 50 nm thick cathode made using the same co-evaporation technique.

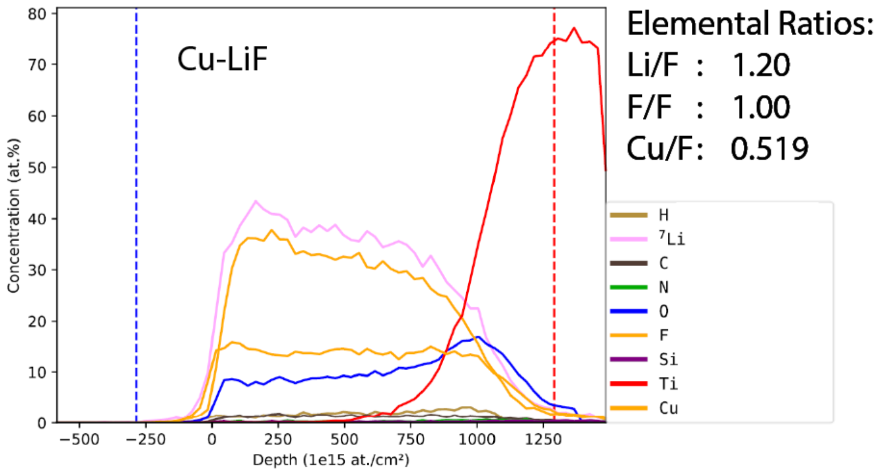

Figure S2: Elastic recoil detection analysis depth profile for the 100 nm thick Cu-LiF cathode. Integrated elemental ratios are also shown. The estimated error for the total elemental ratios is 7%.

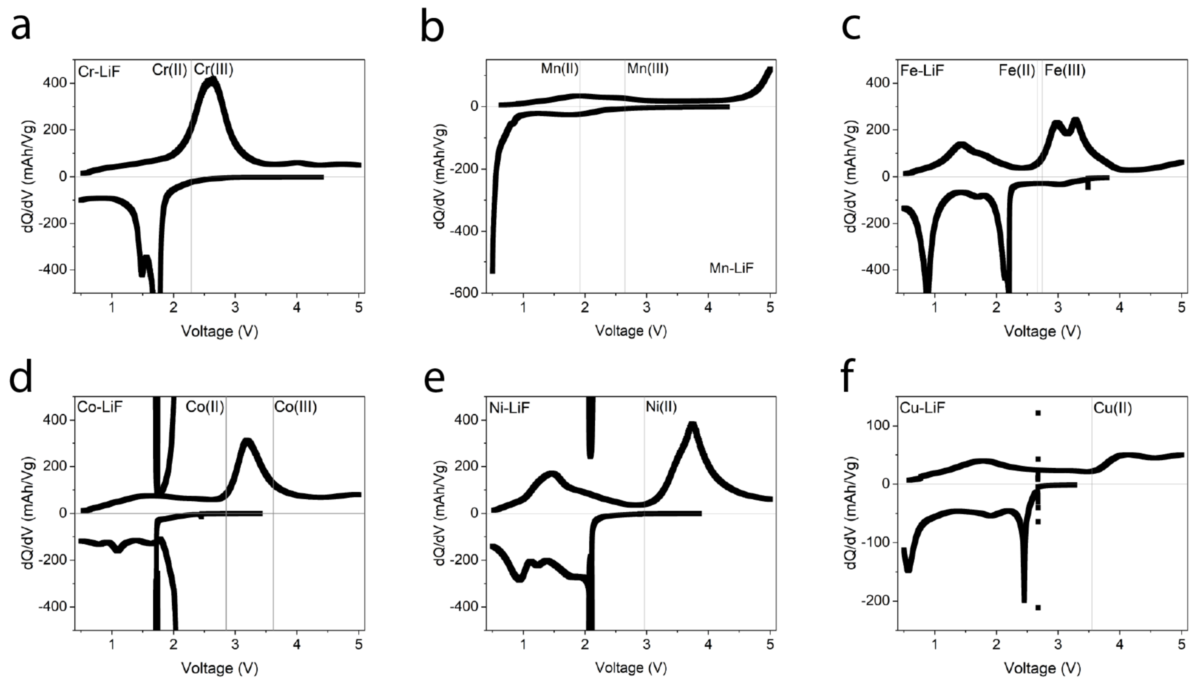

Figure S3: (a) to (f) (Cr to Cu) Calculated dQ/dV vs V curves of all 6 metal cathodes (3rd cycle of Figure 3 of main text). Theoretical bulk EMF values for each corresponding metal reaction are shown as vertical lines.

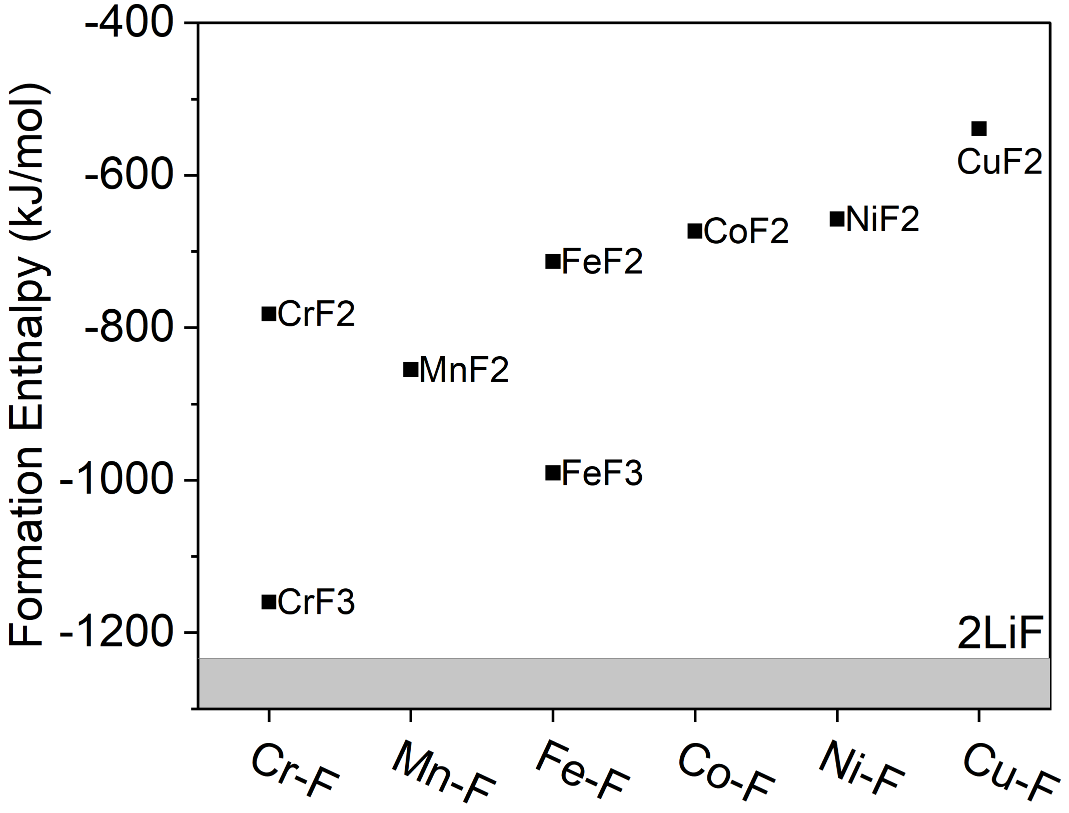

Figure S4: Enthalpies of formation of di- and tri-fluorides of the 6 TMs studied in this work. The horizontal line depicts the formation enthalpy for 2 moles of LiF, which would apply for the reaction enthalpy of the di-fluoride phases.

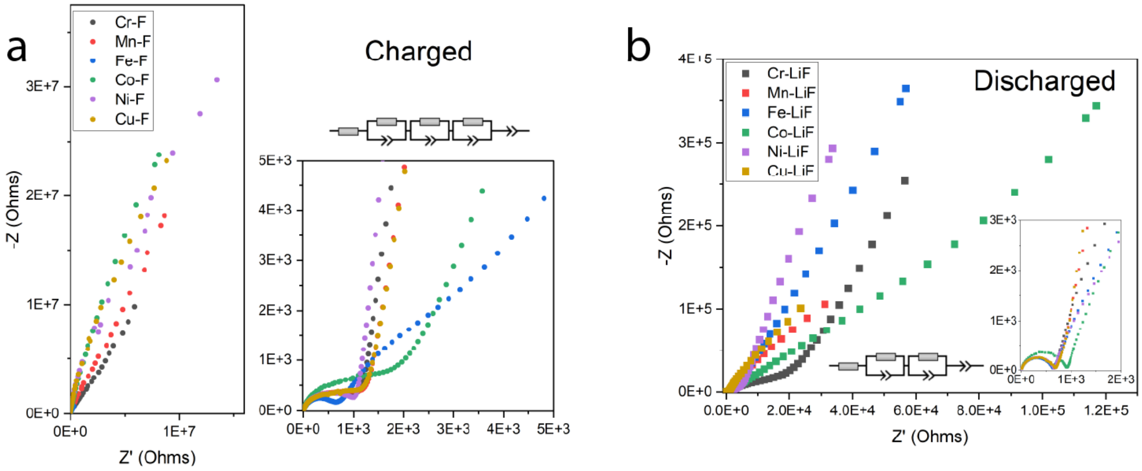

Figure S5: Impedance spectra of all six tested cathodes in (a) charged and (b) discharged states. Cells were charged to 5 V at C/10 followed by a voltage hold at 5 V until the current reached C/100. Cells were discharged to 0.5 V at C/10 followed by a voltage hold at 0.5 V until a current of C/100 was reached. Equivalent circuits used for fitting are shown in (a) and (b) and are different between charged and discharged states as the active material changes phase. These circuits presented the best fitting quality. For conductivity calculations (Figure 6 in main text), the series resistance of the two cathode RC components associated to the cathode is considered.

Table S1: List of DFT runs used for the finetuning of the machine-learned interatomic potential (MLIP). From the runs mentioned in the table, ∼36,000 structures were generated. The finetuning was conducted from a selection of 8,086 structures evenly spaced among all structures (to avoid any biasing).

Formula(s)	Number of Atoms	Type of Run	Temperature (K)
TMF_3 (TM=Cr, Mn, Fe, Co, Ni)	64	Geometry Optimization	0
TMF_2 (TM=Cr, Mn, Fe, Co, Ni, Cu)	24	Geometry Optimization	0
Cr_2F_5	28	Geometry Optimization	0
LiF	215	Geometry Optimization	0
Li_3CrF_6	120	Geometry Optimization	0
Li_2MnF_5	32	Geometry Optimization	0
$LiFe_2F_6$	108	Geometry Optimization	0
$LiFe_2F_6$	108	Geometry Optimization	0
$LiCoF_3$	36	Geometry Optimization	0
TMF_2-Li (TM=Cr, Mn, Fe, Co, Ni, Cu)	49	MD	500
TMF_2-Li (TM=Cr, Mn, Fe, Co, Ni, Cu)	49	MD	150-1500

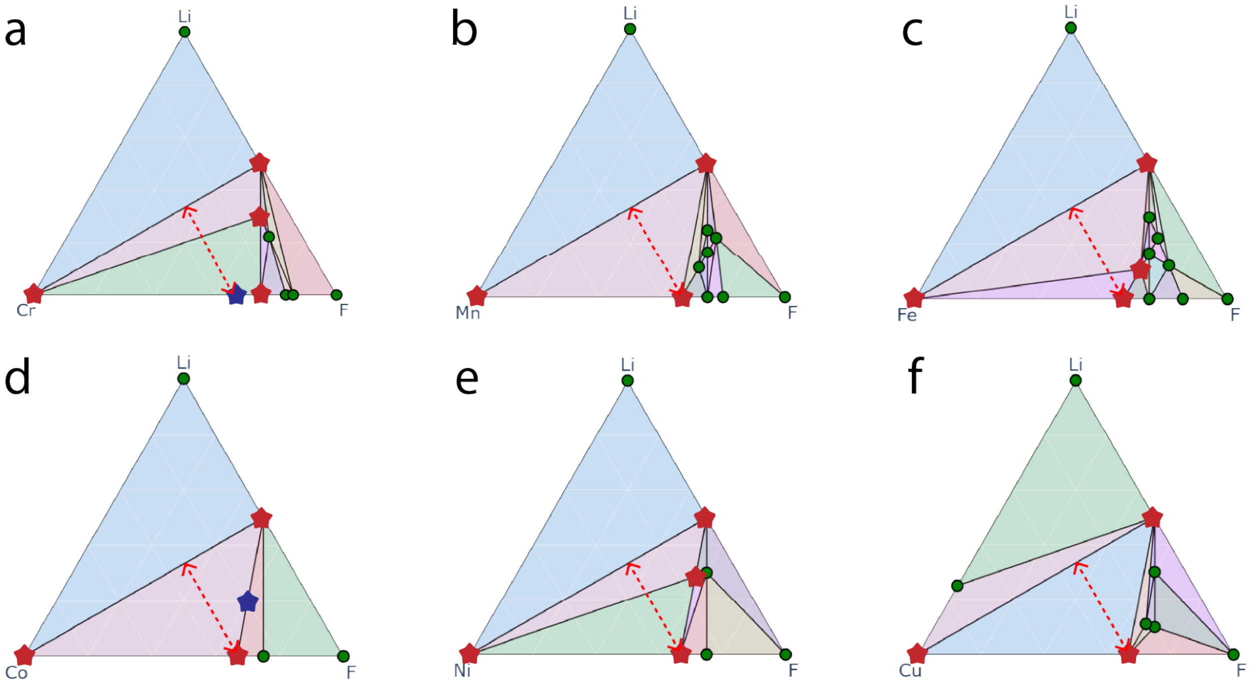

Figure S6: (a) to (f) (Cr to Cu) Predicted phase diagrams of Li-TM-F for TM=Cr, Mn, Fe, Co, Ni, and Cu. Values are taken from material project.[1,2] The blue stars represent a structure not considered stable based on the Materials Project database, but were experimentally observed.[3,4]

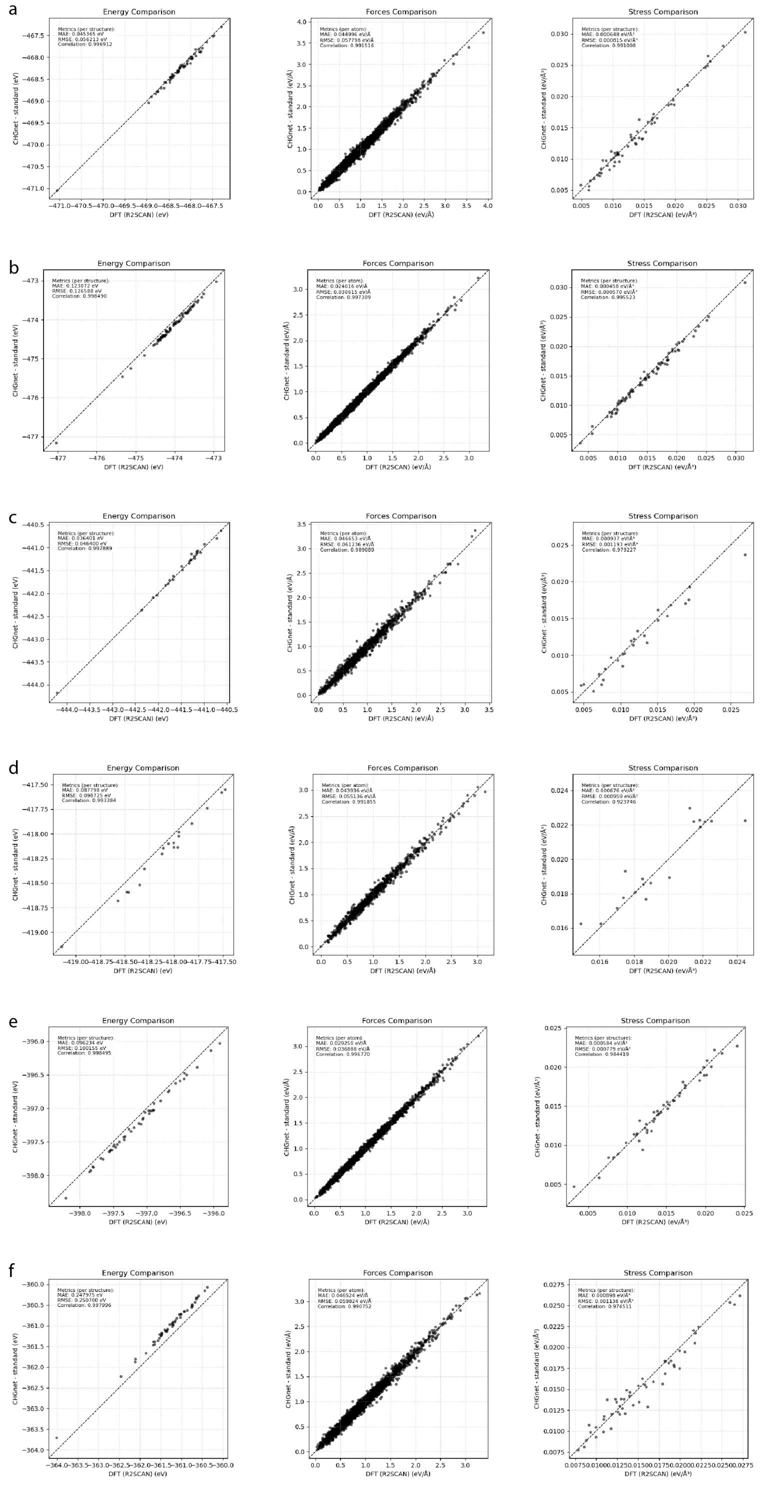

Figure S7: (a) to (f) (Cr to Cu) Benchmarking of finetuned CHGNet graphing neural net model for a selection of DFT calculated structures of Li-Me-F (with Me=Cr to Cu). The benchmarking compares (left to right) energies, forces and stresses of the graphing neural net (y-axis) and DFT (x-axis).

References

(1) Jain, A.; Shyue, .; Ong, P.; Hautier, G.; Chen, W.; William, .; Richards, D.; Dacek, S.; Cholia, S.; Gunter, D.; Skinner, D.; Ceder, G.; Persson, K. A.; Ong, S. P.; Richards, W. D. Commentary: The Materials Project: A materials genome approach to accelerating materials innovation. *APL MATERIALS* **2013**, *1*, 11002.

(2) Ong, S. P.; Wang, L.; Kang, B.; Ceder, G. Li-Fe-P-O 2 Phase Diagram from First Principles Calculations. *Chemistry of Materials* **2008**, *20*, 1798–1807.

(3) Matsuo, Y.; Matsukawa, Y.; Kitakado, M.; Hasegawa, G.; Yoshida, S.; Kubonaka, R.; Yoshida, Y.; Kawasaki, T.; Kobayashi, E.; Moriyoshi, C.; Ohno, S.; Fujita, K.; Hayashi, K.; Akamatsu, H. Topochemical Synthesis of LiCoF 3 with a High-Temperature LiNbO 3-Type Structure. *Inorg. Chem* **2022**, *61*, 11746–11756.

(4) Edwards, A. J. Solid-State Structures of The Binary Fluorides of The Transition Metals. *Advances in Inorganic Chemistry and Radiochemistry* **1983**, *27*, 83–112.
